# Proceedings of the EIP on AHA: A3 Action Group on Frailty

**Published:** 2016-01-31

**Authors:** Maddalena Illario, Guido Iaccarino, Ornella Piazza, Enrica Menditto, Enrico Coscioni

**Affiliations:** 1 Department of Translational Medical Sciences, Federico II University, and R&D Unit, Federico II University Hospital, Italy; 2 Department of Medicine and Surgery, University of Salerno, Italy; 3 CIRFF, Center of Pharmacoeconomics, Federico II University of Naples, Italy; 4 Heart Department, “San Giovanni di Dio e Ruggi D’Aragona” University Hospital, Salerno, Italy

**Keywords:** pensions, health and long-term care, employment, migration and integration, infrastructure development

The present issue of TranslationalMedicine@ UNISA provides a sample of the activities of the partners of the EIP on AHA that are contributing to transform ageing from a burden into an opportunity for the EU, and beyond. It is the expression of a multifaceted approach to a societal challenge that can be only tackled by using inclusive strategy that exploit and valorizes the good practices of multiple stakeholders.

Active ageing is grounded on the concept that older adults full potential can contribute to the well being of society, improving its growth and competitiveness. With this premise, the reading of the present issue of the journal is easier, and its content more coherent. All participants of the European civil society are requested to do their part: from researchers to universities, from public bodies to hospital, from social workers to association of patients, all are requested to bring their contribution to the discussion on how to provide better years to our ageing European citizens. From the reading, it appears that the contribution of research, education and innovation is pivotal. Santulli et al. [2] illustrate the challenge of researchers in order to create models of clinically relevant problems of ageing in order to identify appropriate targets. Indeed, selecting well justified and widely acknowledged model systems represents the best start in designing preclinical studies, especially to overcome any potential bias related to the model itself. This is particularly true in the research that focuses on ageing, which carries unique challenges, mainly attributable to the long lifespan that makes experimental design on human subjects extremely difficult and largely impractical. The authors highlight how more sophisticated animal models of frailty should include a broad range of performance measures in order to properly represent the conditions observed in humans. Unveiling the mechanisms underpinning frailty is essential in developing new clinical therapies, nonetheless the pathway to successful translation of the results requires several careful considerations, including an appropriate choice of animal models, systematic experimental design, and integration of information from both the bench and the bedside.

Ageing is associated with an increase in frail older adults, who are vulnerable and at high risk of adverse health outcomes. Functional impairments often result in falls, immobility and confusion. Early screening for risk of frailty and functional decline in older adults and training of those who start to deploy frail conditions is key to successful prevention. Vuolo et al. [3] show how PERsonalised ICT Supported Services for Independent Living and Active Ageing (PERSSILAA) support early detection of the risk of frailty due to osteoporosis and osteoporosis-related fractures that are growing problems within the ageing population, and are associated with significant morbidity and mortality. Many risk factors have been identified for primary osteoporosis, and the preliminary data from the PERSSILAA population highlights the beneficial effects of primary nutritional interventions, such as the Mediterranean food pattern on bone health.

Adopting a healthy lifestyle is a key aspect for older adults to improve their functional level and delay frailty. A “healthy lifestyle” can be considered the group of steps, actions and strategies that individuals put in place to achieve optimum health. Being physically active along with healthy eating, emotional and spiritual wellbeing are relevant for health. Increasing physical activity is an important strategy to avoid disease and disability, maintain physical and cognitive function and stay engaged in social and productive activities for a successful ageing. PERSSILAA also provided insight on how physical activity can be defined, parameterized and measured in older adults and on different options to deal with citizen physical activity promotion at European level. Among lifestyle factors, nutrition is one of the most important determinants of health, and represents a pivotal element of cancer risk. Di Furia et al. [4] provide an overview of the relationship between several cancers and specific foods and nutrients, highlighting that available data are still inadequate. The working group on lifestyles of the Italian Ministry of Health has developed a comprehensive approach to adequate nutrition, by using a consensus methodology to collect and integrate the available evidences from the literature and from the Italian experiences at the regional level, to outline and scale-up joint strategies for a primary nutritional approach to cancer prevention. Such an approach conjugates surveillance, screening and self-assessment programs with data collection and analysis, offered through a substrate for shared data management, coherently with the Nutrition group of the European Innovation Partnership (EIP) on Active and Healthy Ageing (AHA). The crucial role that diet plays in age-related diseases and cancer is partly mediated by oxidative stress: Conte et al. [5] describe the signalling pathway through which Resveratrol, a polyphenolic antioxidant from grapes, is capable of counteracting the damages caused by oxidation and can induce growth arrest and apoptosis in prostate cancer cells.

Sustainable prevention of frailty through healthy lifestyles conjugates nutrition and physical activity: Corbi et al. [6] provide a review on the knowledge available in literature on the effects of physical activity on cognition and the suggested possible mechanisms involved in these effects. Available evidence suggests that practicing more types of physical activity is particularly advantageous also to cognitive functioning through mechanisms to be further explored.

Guerriero et al. [7] highlighted how important are preventive strategies such as optimization of drug regimen for a better and safer management of elderly patients. The study showed that polypharmacy is more frequent in older patients with a decline in cognitive status, functional status and mobility. All these conditions, associated with polypharmacy regimen can influence frailty status and can affect treatment outcomes with a greater risk for adverse health-related outcomes.

The experiences of the partners of the EIP on AHA suggest that tackling frailty requires innovative approaches integrating multimodal interventions. Multidisciplinarity is the key to sustainable solutions, and ICT tools provide an opportunity: investment in training and education are the key to overcome the difficulties of establishing a common language, and establish durable collaborative practices fostering adoption of the innovations.

The mutual learning between partners of the EIP on AHA has proved productive, and it can transform the good practices into large-scale activities. Importantly, the encounter between A3 organizations and loco-regional, national and international stakeholders is essential to facilitate alignment of objectives and strategies for active and healthy ageing.
